# Prevalence and determinants of depression, anxiety, and burnout among Egyptian house officers during the COVID-19 pandemic

**DOI:** 10.1186/s43045-023-00302-8

**Published:** 2023-03-28

**Authors:** Asmaa Sayed, Marwa Ahmed, Inas El Sayed, Saeed Soliman, Karim Ali, Saged Elsherbiney, Alaaelrahman Shahib, Samar Fares

**Affiliations:** 1 AKasr Alainy Faculty of Medicine, Cairo University, Cairo, Egypt; 2 Cairo University Hospitals, Cairo, Egypt

**Keywords:** COVID-19, House officers, Anxiety, Depression, Burnout

## Abstract

**Background:**

The COVID-19 pandemic is an unprecedented international health crisis, which has invoked massive consequence on healthcare workers’ mental health and wellbeing. This study aimed to detect the prevalence of anxiety, depression, and burnout among house officers amid the COVID-19 pandemic in Egypt to assess the effect of this pandemic on their mental health.

**Results:**

A total of 254 house officers were included in this study, and their responses were analyzed. Anxiety, burnout, and depression were reported among 35%, 32%, and 22% of participants, respectively. Multivariate regression analysis found that higher levels of overall worry were associated with anxiety, but not depression or burnout. Having a good personal protective equipment attitude was a significant predictor of both anxiety and burnout. Participants with depression had a higher likelihood of also having a burnout, but a lower likelihood of having anxiety. Overall worry related to the pandemic, depression, and clinical burnout status were all significantly associated with anxiety.

**Conclusions:**

The study found that anxiety, depression, and burnout are highly prevalent among house officers, who are newly graduated medical professionals. These findings suggest the need for specific programs to address the wellbeing of these individuals during the COVID-19 pandemic.

## Background

The COVID-19 pandemic is considered an unprecedented international health crisis. The highly viral infectivity and severity of COVID-19 have exceeded the capacity of most healthcare systems [1]. This fatal pandemic has a tremendous impact on healthcare workers, especially the frontlines young doctors. They fear catching the infection by themselves or tainting their families. Moreover, the pressure of increased workload, no rest, inadequate eating, no affordability of personal protective equipment, and working with frequently changing new protocols are obstacles healthcare workers must face. Also, the rapid spread of the disease to medical staff and caring for their colleagues also for critically ill rapidly deteriorating patients are heavy loads upon them [2–5].

Most healthcare workers have no alternative caregiver for their families through their quarantine. They are concerned about infecting their families [6]. In addition, healthcare workers realize that being young does not guarantee them protection against COVID-19 [7].

All these stressors if cannot be coped with make healthcare workers more vulnerable to psychological disorders amid this epidemic [8]. House officers are in an intermediate stage between undergraduate and practice of medicine. They experience many stressors and are more exposed to psychological manifestations such as stress, anxiety, burnout, and depression [9]. Moreover, the prevalence of anxiety, burnout, and depression highly increases in disasters [10].

Burnout syndrome is a risky overload state that could be complicated by multiple physical or mental illness [11]. It was first detected in the early 1970s, especially among healthcare providers [12]. Occupational burnout remains a critical risk factor affecting the quality of life and health of healthcare workers, particularly frontline defensers [13].

Burnout causes detraction from optimal working capacities. It is experienced by the individual with a high level of physical, emotional, and psychological fatigue [14]. The main core of burnout is exhaustion and fatigue. Burnout has many dramatic drawbacks; it causes an increase in medical errors, a reduction in professional performance, and higher rates of absenteeism, a decrease in job satisfaction, an increase in medical leave, and more personal suffering [15].

Good organizational support must be afforded. The protection of frontlines young physicians should be a priority and policymakers should make evidence-based decisions [16]. As the COVID-19 pandemic affects the psychological status of healthcare workers negatively, it affects the quality of medical care negatively [17]. The COVID-19 pandemic is represented as a long-distance race. We should support our labor force to keep on providing the standard medical services [18].

Despite many calls for mental health support for healthcare workers through the COVID-19 pandemic being released [19, 20], to our knowledge, no study on mental health disorders among house officers has been reported. Hence, there is a dire need to investigate and assess the psychological effects of the COVID-19 pandemic on house officers and define risk and protective factors. The aim of this study is, then, to investigate the prevalence of depression, anxiety, and burnout among house officers amid the COVID-19 pandemic in Egypt, gauging the effect of such pandemic on their mental health.

## Methods

### Study design and participants

This cross-sectional study was conducted between March 3, 2020, and June 17, 2020. Due to the COVID-19 pandemic, it was not applicable to interview the house officers in person. Therefore, a Google form was designed and uploaded on different blogs such as Facebook groups, in which house officers, who were spending their training in non-isolation departments and outpatient clinic of teaching hospitals of different universities in Egypt, can find and fill out.

#### Sample

The sample size for this cross-sectional survey was calculated using the CDC Epi info ® version 7 software on the following assumptions: anticipated frequency of self-reported depression, anxiety was 13% [21] with an error margin of 5%, and 95% confidence interval; the required sample size was 174 participants. For burnout, the expected prevalence is 22% with an error margin of 5% and a 95% confidence interval; the required sample size was 251. So, the actual collected sample was 254.

### Data collection

All participants reported their demographic data, COVID-19-related information, and completed three standardized questionnaires which assessed their depressive symptoms, anxiety disorder, and burnout syndrome. Finally, a total of 254 house officers who completed the questionnaires were included in the analysis.

### Ethical statement

This study was conducted in accordance with the Declaration of Helsinki. Electronic informed written consent was obtained from each participant prior to starting the survey. Participants had the opportunity to withdraw from the survey before submission without providing any justification. It was completely voluntary and noncommercial.

### Measures

#### Demographic information

Demographic variables included gender (male or female), age, residence (Cairo, outside Cairo), marital status (single or married), and training hospital (Cairo University or others).

#### COVID-19-related exposures

This section was evaluated in terms of four items: (1) contact with a COVID-19 case, which might also lead to infection, (2) personal high risk of severe COVID-19, (3) family member at high risk of severe COVID-19, and (4) participants’ self-reported overall worry regarding COVID-19 on 1–5 scale.

#### Anxiety disorder

The current study used 21-item Beck Anxiety Inventory (BAI) [22] to assess house officer’s anxiety symptoms. It measures the severity of anxiety and associated emotional, physiological, and cognitive symptoms. Each of the BAI items is rated on 4 scales with four possible answer choices:

- Not at all
- Mildly (It did not bother me much)
- Moderately (It was very unpleasant, but I could stand it)
- Severely (I could barely stand it).

A value (from 0 to 3) was then assigned to each response where zero represents not at all and 3 represents severely. The total score for all 21 symptoms ranged from 0 to 63 points. A total score of 0–7 was interpreted as a “minimal” level of anxiety; 8–15 as “mild”; 16–25 as “moderate,” and 26–63 as “severe.” In this way, the moderate and severe were considered as having anxiety.

*The BAI is copyrighted by and currently available from Pearson Education, Inc. (* *http://www.pearsonassess.com**).*

#### Depressive symptoms

The 9-item patient health questionnaire-9 (PHQ-9) was used to identify whether the house officer had depressive symptoms and assess the frequency of depressive symptoms over the past 2 weeks on a 9-point (PHQ-9) scale ranging from 0 (not at all) to 3 (nearly every day). The score range of the PHQ-9 was 0–27 points, and higher scores range from 20 to 27 indicating severe depressive symptoms. In this context, the moderate and severe were considered as having depression.

### Burnout symptoms

The Maslach Burnout Inventory (MBI) was used to assess burnout. It was first described by Maslach & Jackson in 1981 [23]. It is a psychological assessment tool consisting of 22 symptom items [24]. It takes about 10 min to complete filling the MBI [22]. In the MBI, the three dimensions of burnout—emotional exhaustion, personal accomplishment measures, and depersonalization—are measured and validated [24].

There are five versions of the MBI: Human Services Survey (MBI-HSS), Human Services Survey for Medical Personnel (MBI-HSS (MP)), Educators Survey (MBI-ES), General Survey (MBI-GS), and General Survey for Students (MBI-GS [S]) [24]. In this study, Human Services Survey (MBI-HSS) was used. It consists of 22 items. It is the most commonly used version of the MBI [25].

The items of MBI are scored on a 7-Likert score ranging from “never” (0) to “daily” (6). It has three section scales: emotional exhaustion (9 items), personal achievement (8 items), and depersonalization (5 items). Each scale assesses its own section of burnout, separately. Scales should not be combined to form a single burnout scale. In this work, the participant is considered clinically burnout if he has high emotional exhaustion with either high depersonalization or low personal accomplishment.

The 7-Likert score of MBI are as follows:

- Never (0)
- A few times a year or less (1)
- Once a month or less (2)
- A few times a month (3)
- Once a week (4)
- A few times a week (5)
- Every day (6)

### Statistical analysis

Using Stata ® version 16 software, descriptive statistics were provided to describe and summarize data by numbers and percentages and bivariate analysis was employed to examine associations of outcome variables with independent variables. The Rao-Scott chi-square test statistic (*χ*^2^) was used to cross-tabulate depression, anxiety, and burnout status by covariates of gender, residence, marital status, training hospital, contact with COVID-19 cases, personal and family risk of severe COVID-19, and overall worry from COVID-19. Multivariate logistic regression analysis was conducted to determine the association between anxiety, depression, and burnout status and covariates. Covariates which were entered in the model were those found to be significantly associated with anxiety, depression, and burnout in the bivariate analysis (two-tailed *P* value<0.05 was considered statistically significant).

## Results

A total of 254 house officers were included in this study, and their responses were analyzed. Table 1 shows the basic demographic characteristics, COVID-19 disease risk, and exposures as well as overall worry about the pandemic. The mean (± SD) age was 25 (±1.0) years. More than half of the participants were females (53.9%, *n*= 137). Most participants reside in Cairo (78.3%, *n*=199), are not married (94.5%, *n*=240), had direct contact with COVID-19 cases (83.5%, *n*=212), do not have risk factors for severe COVID-19 disease (88.6%, *n*=212), and do live with a family member that is at high risk for severe COVID-19 disease (73.6%, *n*=187). On a scale of one to five, their mean (±SD) score of worry regarding the pandemic was 3.5±1.0. Lastly, only 16.3% (*n*=43) of them showed a willingness to participate in the management of COVID-19 cases, while 41.7% (*n*=106) said they are not willing.

**Table 1:** Characteristics of the study population (*N*= 254)

	Total	% of total
**Age (mean, SD)**	25.0, 1	
**Gender**		
Females	137	53.94
Males	117	46.06
**Residence**		
Cairo	199	78.35
Others	55	21.65
**Marital status**		
Single	240	94.49
Married	14	5.51
**Had direct contact with COVID cases**		
Yes	212	83.46
No	42	16.54
**Personal high risk for severe COVID**		
Yes	29	11.42
No	225	88.58
**Living with family members at high risk for severe COVID**		
Yes	187	73.62
No	67	26.38
**Overall worry about COVID-19 pandemic (Mean, SD)**	3.46, 1	
1	10	3.95
2	31	12.25
3	94	37.15
4	69	27.27
5	49	19.37
**Willingness to participate in management of COVID cases**		
Yes	43	16.93
No	106	41.73
May be	105	41.34

**Fig. 1:**
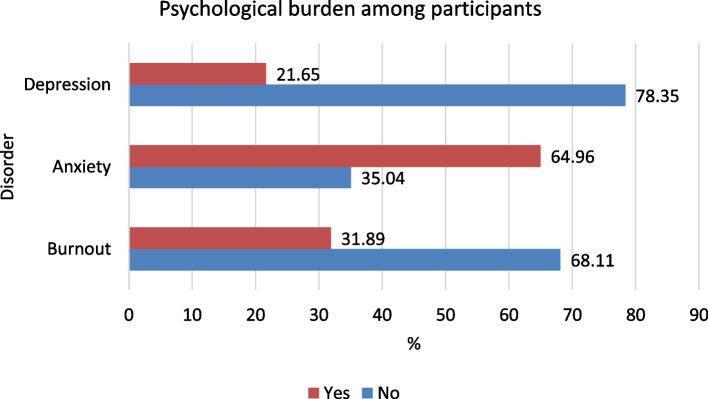
Prevalence of depression, anxiety, and burnout among Egyptian house officers amid the COVID-19 pandemic

Regarding the psychological burden among the people study participants, anxiety, burnout, and depression occurred among 35.0%, 31.9%, and 21.7% of participants, respectively, as illustrated in Fig. 1.

The associations of depression were presented. Gender, residence, and marital status were not associated with depression occurrence. Whereas directly contacting COVID-19 cases was significantly associated with depression (*P =* 0.012). Depression was reported among 24.53% of COVID-19-exposed house officers versus only 7.1% of non-exposed. Having a personal risk for severe COVID-19 was not associated with depression; however, having a family member with that risk was a significant factor with the prevalence of 25.13% (*P=* 0.024). Also, the overall worry related to the pandemic was associated with depression occurrence among the participants. Although preparedness was not linked to the depression status, the PPE attitude was significantly linked to it (*P=* 0.036). Also, anxiety and burnout status were highly significant factors for the depression status (*P* < 0.001), as shown in Table 2.

**Table 2:** Bivariate association between characteristics of the study population and depression (*N*=254)

	Not depressed		Depressed			
	***N***	%	***N***	%	P*	***X***^**2^**^
**Gender**						
Female	106	77.37	31	22.63	0.683	0.17
Male	93	79.49	24	20.51		
**Residence**						
Cairo	155	77.89	44	22.11	0.838	0.35
Others	44	81.48	11	20.37		
**Marital status**						
Single	187	77.92	53	22.08	0.491	0.47
Married	12	85.71	2	14.29		
**Had direct contact with COVID-19 cases**						
No	39	92.86	3	7.14	0.012	6.24
Yes	160	75.47	52	24.53		
**Personal high risk for severe COVID-19**						
No	177	78.67	48	21.33	0.730	0.12
Yes	22	75.86	7	24.14		
**Living with family members at high risk for severe COVID-19**						
No	59	88.06	8	11.94	0.024	5.06
Yes	140	74.87	47	25.13		
**Overall worry about COVID-19 pandemic**						
1	7	70.00	3	30.00	0.022	11.46
2	28	90.32	3	9.68		
3	79	84.04	15	15.96		
4	53	76.81	16	23.19		
5	31	63.27	18	36.73		
**Preparedness**						
Bad	136	75.14	45	24.86	0.051	3.82
Good	63	86.30	10	13.70		
**Personal protective equipment attitude**						
Bad	33	91.67	3	8.33	0.036	4.39
Good	166	76.15	52	23.85		
**Has anxiety**						
No	47	52.81	42	47.19	<0.001	52.67
Yes	152	92.12	13	7.88		
**Clinically Burnt out**						
No	152	87.86	21	12.14	<0.001	28.95
Yes	47	58.02	34	41.98		

*P value is considered significant if <0.05

^^^*X*^2^ Pearson chi-square test

Regarding the factors related to anxiety status among the participants, only the overall worry related to the pandemic, depression, and clinical burnout status was significantly related to anxiety among the participants (*P* < 0.001), as illustrated in Table 3.

**Table 3:** Bivariate association between characteristics of the study population and anxiety (*N*=254)

	No anxiety		Anxiety			
	***N***	%	***N***	%	P*	***X***^**2^**^
**Gender**						
Female	53	38.69	84	61.31	0.187	1.74
Male	36	30.77	81	69.23		
**Residence**						
Cairo	71	35.68	128	64.32	0.335	2.19
Others	18	33.33	37	66.67		
**Marital status**						
Single	84	35.00	156	65.00	0.957	0.01
Married	5	35.71	9	64.29		
**Had direct contact with COVID-19 cases**						
No	12	28.57	30	71.43	0.336	0.92
Yes	77	36.32	135	63.68		
**Personal high risk for severe COVID-19**						
No	75	33.33	150	66.67	0.112	2.52
Yes	14	48.28	15	51.72		
**Living with family members at high risk for severe COVID-19**						
No	18	26.87	49	73.13	0.102	2.67
Yes	71	37.97	116	62.03		
**Overall worry about COVID-19 pandemic**						
1	2	20.00	8	80.00	<0.001	38.41
2	3	9.68	28	90.32		
3	20	21.28	74	78.72		
4	34	49.28	35	50.72		
5	30	61.22	19	38.78		
**Preparedness**						
Bad	70	38.67	111	61.33	0.056	3.65
Good	19	26.03	54	73.97		
**Personal protective equipment attitude**						
Bad	12	33.33	24	66.67	0.817	0.05
Good	77	35.32	141	64.68		
**Has depression**						
No	47	23.62	152	76.38	<0.001	52.67
Yes	42	76.36	13	23.64		
**Clinically Burnt out**						
No	42	24.28	131	75.72	<0.001	27.60
Yes	47	58.02	34	41.98		

*P value is considered significant if <0.05

^^^*X*^2^ Pearson chi-square test

As regards the bivariate associations with burnout among the participants, gender, residence, marital status, and personal or family risk to severe COVID-19 were not significantly related to the burnout status of the participants. Directly contacting COVID cases was significantly associated with burnout with the prevalence of 34.91% among those participants (*P =* 0.020). Overall worry, personal and institutional preparedness were not linked to the burnout status of the participants, while PPE attitude, depression, and anxiety status were significant factors for burnout (*P* < 0.001), as displayed in Table 4.

**Table 4:** Bivariate association between characteristics of the study population and burnout (*N*=254)

	Not burnt out		Clinically burnt out			
	***N***	%	***N***	%	P*	***X***^**2^**^
**Gender**						
Female	96	70.07	41	29.93	0.468	0.53
Male	77	65.81	40	34.19		
**Residence**						
Cairo	132	66.33	67	33.67	0.440	1.64
Others	41	75.93	14	24.07		
**Marital status**						
Single	162	67.50	78	32.50	0.388	0.75
Married	11	78.57	3	21.43		
**Had direct contact with COVID-19 cases**						
No	35	83.33	7	16.67	0.020	5.37
Yes	138	65.09	74	34.91		
**Personal high risk for severe COVID-19**						
No	154	68.44	71	31.56	0.750	0.10
Yes	19	65.52	10	34.48		
**Living with family members at high risk for severe COVID-19**						
No	48	71.64	19	28.36	0.470	0.52
Yes	125	66.84	62	33.16		
**Overall worry about COVID-19 pandemic**						
1	6	60.00	4	40.00	0.147	6.80
2	26	83.87	5	16.13		
3	66	70.21	28	29.79		
4	46	66.67	23	33.33		
5	28	57.14	21	42.86		
**Preparedness**						
Bad	122	67.40	59	32.60	0.703	0.14
Good	51	69.86	22	30.14		
**Personal protective equipment attitude**						
Bad	33	91.67	3	8.33	0.001	10.72
Good	140	64.22	78	35.78		
**Has depression**						
No	152	76.38	47	23.62	<0.001	28.95
Yes	21	38.18	34	61.82		
**Has anxiety**						
No	42	47.19	47	52.81	<0.001	27.60
Yes	131	79.39	34	20.61		

^*^P value is considered significant if <0.05

^^^*X*^2^ Pearson chi-square test

Multivariate regression analysis for the occurrence of depression and anxiety and burnout among study participants was done and revealed, higher levels of overall worry score predicted anxiety but not depression or burnout, and paradoxically, a good personal protective equipment attitude was a significant predictor for anxiety (OR 2.67, CI 1.03–6.9) and burnout (OR 6.1, CI 1.2–5.2). Participants with depression had a higher odd for having burnout (OR 2.48, CI 1.18–5.190) but lower odds for anxiety (OR 0.3, CI 0.15–0.60), as shown in Table 5.

**Table 5:** Multivariate regression analysis for depression, anxiety, and burnout predictors among Egyptian house officers amid the COVID-19 pandemic

	Depression		Anxiety		Burnout	
	OR (95% CI)^**a**^	***P***	OR (95% CI)	***P***	OR (95% CI)	***P***
**Had direct contact with COVID-19 cases**						
Yes	3.75 (0.91–15.50)	0.068	1.31 (0.55–3.15)	0.540	2.00 (0.79–5.09)	0.146
**Family members at high risk for severe COVID**						
Yes	2.09 (0.82–5.28)	0.120	0.83 (0.39–1.76)	0.629	0.90 (0.45–1.81)	0.776
**Overall worry about COVID-19 pandemic**						
2	0.26 (0.03–.98)	0.195	0.83 (0.07–9.41)	0.878	0.33 (0.06–1.74)	0.191
3	0.27 (0.05–1.43)	0.123	0.33 (0.04–2.73)	0.305	0.57 (0.14–2.34)	0.435
4	0.19 (0.03–1.08)	0.061	0.07 (0.01–0.62)	0.017	0.38 (0.09–1.67)	0.202
5	0.35 (0.06–1.99)	0.235	0.06 (0.01–0.59)	0.014	0.55 (0.12–2.47)	0.433
**Personal protective equipment attitude**						
Good	3.54 (0.82–15.33)	0.091	2.67 (1.03–6.89)	0.042	6.19 (1.67–22.92)	0.006
**Depression**						
Yes	-	-	0.11 (0.05–0.25)	<0.001	2.48 (1.18–5.19)	0.016
**Anxiety**						
Yes	0.11 (0.05–0.26)	<0.001	-	-	0.30 (0.15–0.60)	0.001
**Burnout**						
Yes	2.51 (1.20–5.25)	0.014	0.28 (0.14–0.58)	0.001	-	-

^a^*OR* odds ratio, *CI* confidence interval

## Discussion

Embarking on a new chapter of their medical career, novice HCWs, especially house officers, are likely intimidated by aggravation and uncertainties in normal days; for those house officers starting their medical career in 2020 with the unprecedented, enigmatic, international health emergency (COVID-19), there were multiple stressors that they encountered as HCWs are frontline warriors against the mysterious virus. On an administrative and managerial level, it is crucial to address these issues to provide support and motivation in order to alleviate the mental and psychological implications of HCWs [26].

The current study was aimed to investigate and assess the psychological effects of the COVID-19 pandemic on house officers and define risk and protective factors. So, the prevalence of depression, anxiety, and burnout among house officers amid the COVID-19 pandemic in Egypt was assessed, gauging the effect of such pandemic on their mental health.

The results of this study showed that more than one third of the house officers had anxiety, thus confirming the multiple stressors facing the health professionals. This prevalence proportion exceeds the researchers’ expectations based on reported previous studies such as the study conducted in China by Zhang et al. to compare the psychological problems among medical and non-medical health workers during the pandemic, and the level of anxiety was 13% among medical health workers [27]. Also, in the meta-analysis conducted by Pappa et al. in 2020 for 12 studies to assess the prevalence of anxiety among healthcare workers during the COVID-19 pandemic, the pooled prevalence was 23.21% (95% CI 17.77–29.13) [28]. Only approximating results from China including the Wuhan region where the first strike of the virus occurred reported an anxiety prevalence of 45% [1] Egypt though had time and opportunity for psychological preparedness as the first case of COVID-19 was detected on the 15th of February, and the community spread phase started late in May 2020 according to WHO Regional Office of Eastern Mediterranean reports [29].

The high prevalence of anxiety among the house officers in this study can be attributed partially to their young age and lack of experience and training in a new setting for newly graduated doctors given the unintentional lack of knowledge of the nature of the COVID-19 pandemic [30–32].

Another explanation is the nature of house officers’ training: they are required to rotate in different specialties in the hospital which put them in direct contact with a wider number of patients that may increase the risk of infection. Also, working for extended hours with the constant need for personal protective equipment (PPE) increases their physical fatigue which may negatively affect their mental health [32].

Unlike in previous pandemics, in the COVID-19 pandemic, the media circulation today is way easier; hence, the house officers are continuously exposed to and bombarded with news, experiences, information from all over the world [33], and the news of colleagues’ infections and death as 103 doctors deceased suffering from COVID-19 infection in Egypt until 9th of July 2020 as reported by the Egyptian Medical Syndicate [34].

The depression prevalence proportion among the respondent house officers was 21.7%; such finding was in line with that of the study conducted in Italy by Rossi et al. with a depression prevalence of 19.8% [30]. However, anxiety, depression, and burnout levels were not correlated to gender, residency, marital status, and personal risk for severe COVID-19 infection.

House officers with family members at high risk of severe COVID-19 were more vulnerable to anxiety, depression, and burnout. Fear of infecting one’s own family was one of the major stressors facing HCWs, and many were obliged to stay away from family depriving the HCWs from a coping method and the needed family support [26]. On the other hand, the correlation between the personal risk of severe COVID-19 and anxiety, depression, or burnout was not significant which may reflect their professional commitment to their duty regardless of the risk; almost half of the house officers had no objection to deal with and treat COVID-19 patients.

House officers with training outside Cairo University Hospitals, have a three-fold increase in the likelihood of being depressed (OR, 3.01; 95% CI, 1.02, 8.83; *P*=0.045) which can be justified by lack of PPE, lack of organizing roles, and shortage of healthcare providers.

The increasing overall worry scale was associated with the increasing odds of developing depression. For anxiety, this study showed an interesting finding: the majority of house officers with anxiety rated their level of overall worry with the least levels on the scale. This could be a means of coping with their hidden stress, a denial, an inner fear related to stigma, or stubbornness to call for help. This calls for further in-depth studies to analyze such phenomenon.

Physician’s burnout is an international public health problem; unfortunately, the COVID-19 pandemic has aggravated physical stressors on the frontline physicians leading to increase their burnout [35]. Unfortunately, healthcare providers suffered extra pressure during that pandemic to match between their family duties and their responsibilities towards their patients [36].

The results of this study showed that more than one third of the studied physicians (31.9%) had burnout during the COVID-19 pandemic. These results were matched but less than the results of a medical economics staff which revealed that about two thirds of participants (65%) reported that their feeling of burnout has been increased after the COVID-19 pandemic in 2020 [37]. It can be explained by, apprehensions of being contaminated with COVID-19 or tainting their families during the pandemic, increased workload with longer work hours, shortages of PPE, deficient coping mechanisms, or support amid the pandemic [38].

This study results showed a significant association between burnout and the risk of dealing with confirmed COVID-19 cases. This is matched with the study conducted by McHill et al. [39] who reported that the more exposure to COVID-19 positive cases, the more exposure of burnout, most probably due to reduced rest time and sleep.

The current study strongly addresses distress among house officers despite some limitations that emerged during conducting this study. For instance, it is an observational study with an online survey which does not provide us with the response rate and was based on self-assessment; besides, the fact that according to the retrieved data, also it does not evaluate the mental state of the participants by clinical examinations or identify the vulnerabilities for anxiety and depression in this cohort of house officers using logistic regression analysis indicates that need for further studies on larger scales to identify the risk factors.

The mental well-being of health professionals should be one of the top priorities of healthcare executives in order to timely provide innovative strategies to alleviate the psychological burden. The current literature has suggested many solutions as hotlines, coping mechanisms, training, and effective leadership in addition to a high quality of training and availability of adequate equipment during such pandemics, emphasizing the effect of peer communication and support [11]. While efforts to prevent stress among HCWs or even reduce its occurrence by promoting positive, adaptive ways to face the ongoing challenges may seem impossible, the experience gained from this pandemic suggests that we can successfully enhance our resilience.

## Conclusions

Psychological implications of the house officers as freshly graduated doctors starting their medical career in the era of COVID-19 are associated with highly prevalent burnout, anxiety, and depression. The findings of this study stress the dire need to support young healthcare professionals to relieve the negative psychological impacts of that pandemic. Also, setting preventive and coping strategies with early detection and proper management of these problems in the work atmosphere is a mandatory.
